# Dr. A .A. Mantell

**Published:** 1911-09-09

**Authors:** 


### OBITUARY.

The death is announced of Dr. A .A. Mantell on August 22nd at Bathampton, Bath, at the age of eighty. He was formerly surgeon-major in the Indian Army.

